# SUMOylation as a Therapeutic Target for Myocardial Infarction

**DOI:** 10.3389/fcvm.2021.701583

**Published:** 2021-07-28

**Authors:** Wei Zhao, Xiuying Zhang, Jianhui Rong

**Affiliations:** ^1^Li Ka Shing Faculty of Medicine, School of Chinese Medicine, University of Hong Kong, Hong Kong, China; ^2^Zhujiang Hospital, Southern Medical University, Guangzhou, China; ^3^Shenzhen Institute of Research and Innovation, The University of Hong Kong, Shenzhen, China

**Keywords:** myocadial infarction, sumoylation, sumoylated proteins, small ubiquitin-like modifier, myocardial ischemia-reperfusion injury

## Abstract

Myocardial infarction is a prevalent and life-threatening cardiovascular disease. The main goal of existing interventional therapies is to restore coronary reperfusion while few are designed to ameliorate the pathology of heart diseases via targeting the post-translational modifications of those critical proteins. Small ubiquitin-like modifier (SUMO) proteins are recently discovered to form a new type of protein post-translational modifications (PTM), known as SUMOylation. SUMOylation and deSUMOylation are dynamically balanced in the maintenance of various biological processes including cell division, DNA repair, epigenetic transcriptional regulation, and cellular metabolism. Importantly, SUMOylation plays a critical role in the regulation of cardiac functions and the pathology of cardiovascular diseases, especially in heart failure and myocardial infarction. This review summarizes the current understanding on the effects of SUMOylation and SUMOylated proteins in the pathophysiology of myocardial infarction and identifies the potential treatments against myocardial injury via targeting SUMO. Ultimately, this review recommends SUMOylation as a key therapeutic target for treating cardiovascular diseases.

## Introduction

Cardiovascular diseases have recently become a leading contributor to the burden to the global health care systems and economy (1). Myocardial infarction (MI) jeopardizes the health and life of more than 7 million individuals worldwide each year, ~550,000 first episodes and 200,000 recurrent episodes (2). Upon MI, sudden ischemia causes myocardial necrosis, heart failure, and even cardiac arrest (3). The existing interventional coronary reperfusion strategies are effective in controlling morbidity and mortality of MI. However, novel therapies are pressingly needed to increase the survival rate and reduce the re-occurrence of MI. The current effort is directed to the development of new drugs for the treatment of heart failure (4). On the other hand, effective medicines are also needed to halt disease progression and promote the recovery of cardiac functions.

It is well-known that some proteins fail to function properly and others become hyperactivated in heart diseases due to the dysregulation of protein post-translational modifications (PTM) including phosphorylation, acetylation, glycosylation, amidation, hydroxylation, methylation, ubiquitylation and sulfation (5, 6). These PTMs are dynamically balanced by conjugation and de-conjugation by functionally opposing enzymes. Indeed, PTMs are crucial for adapting various signaling pathways to maintain cellular homeostasis and adapt the cells to various stress stimuli (7).

**Figure 1:**
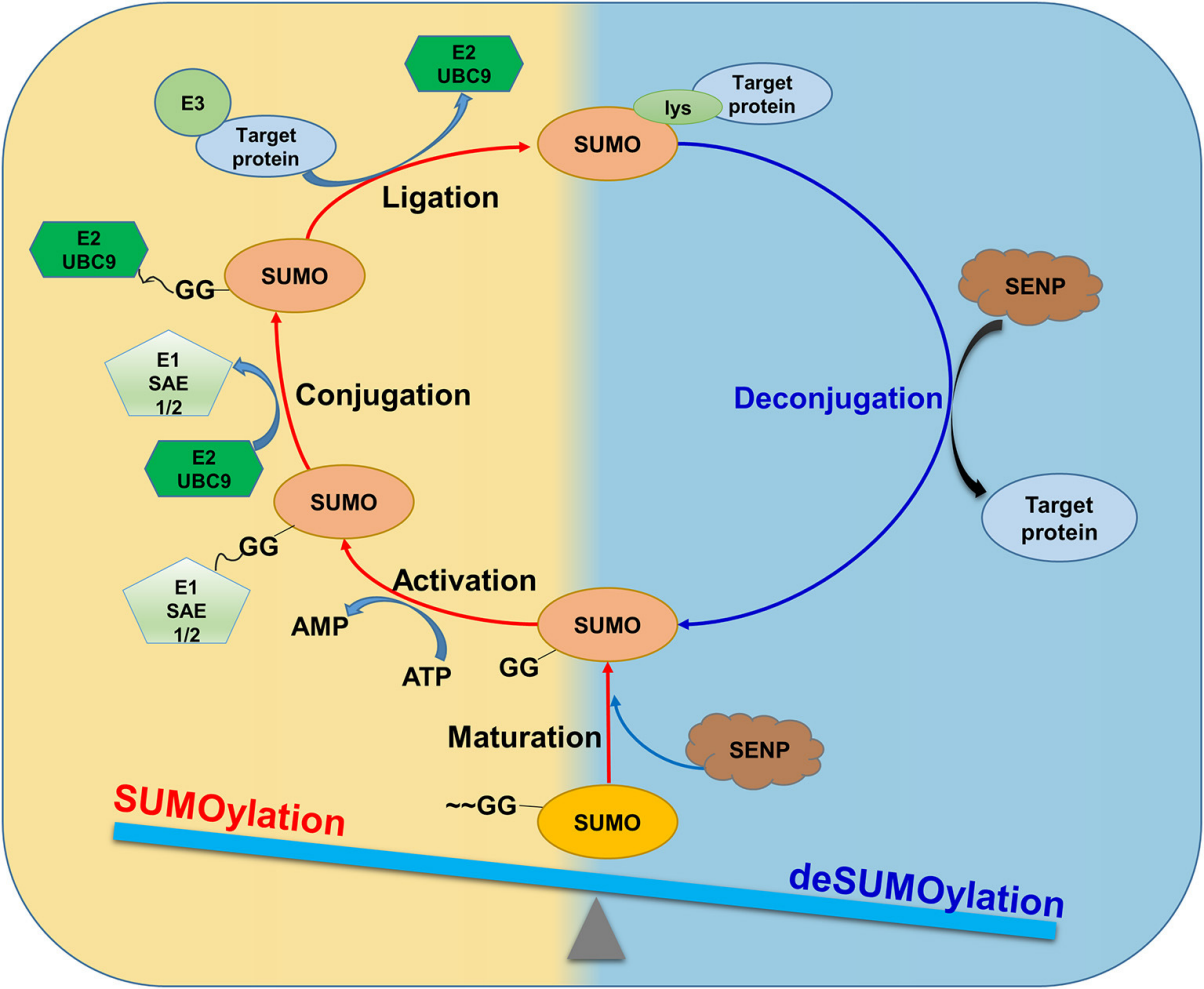
SUMOylation and deSUMOylation are dynamically balanced under physiological conditions. SUMO proteins are proteolytically cleaved by SUMO/ SENP to form mature forms. The matured SUMOs are activated by SAE1/SAE2 in an ATP-dependent manner. E2 UBC9 catalyzes the transesterification to form E2 UBC9 conjugate while the SUMO E3 ligase catalyzes the SUMOylation by transferring SUMOs to the target protein at the specific lysine residues. On the other hand, the SUMOylated proteins undergo deconjugation to dissociate the target protein-SUMO conjugates by SENP. Under physiological conditions, SUMOylation and deSUMOylation are dynamically balanced.

Among different PTMs, SUMOylation describes that a family of five small ubiquitin-like modifier (SUMO) proteins, namely SUMO1-5, form covalent and reversible conjugates with selected proteins and thereby regulates various cellular processes and functions (8, 9). During the SUMOylation, the SUMO proteins are proteolytically cleaved by SUMO/sentrin-specific protease (SENP) to yield the mature form of SUMOs. Subsequently, the SUMO E1 enzyme (SAE1/SAE2) activates the matured SUMOs while the SUMO E2 conjugating enzyme (UBC9) catalyzes the conjugation. Furthermore, the SUMO E3 ligase catalyzes the SUMOylation by transferring SUMOs to the target proteins. On the other hand, the SUMOylated proteins undergo deconjugation by SENP via dissociating the target protein-SUMO conjugates. Under physiological conditions, SUMOylation and deSUMOylation are dynamically balanced as shown in Figure 1. By contrast, the imbalance between SUMOylation and de-SUMOylation is implicated in cardiac diseases (10–12). Previous studies focused on the role of SUMOylation in heart failure (8, 13). For instance, a small molecule N106 was used to target SERCA2a SUMOylation as a potential treatment for heart failure (8). Interestingly, exosomes were isolated from fibroblasts in heart failure and might deliver miR-146 to reduce SUMO-1 expression in cardiac myocytes (14). However, the roles of SUMOylation in MI have not been fully examined.

This review aims to update the current knowledge on the role of SUMOylation and SUMOylated proteins in the pathology and cardiac repair of MI. Previous studies have reported some SUMOylation proteins as targets for the treatment of MI (15–17). Thus, this review discussed the effects of the SUMOylation on the subcellular location of these protein targets and the progression of MI and overview the functions and clinical application stages of protein SUMOylation. We would highlight the SUMO machinery as a potential therapeutic target for drug discovery against MI injury.

## Process of Protein SUMOylation

### The Family of SUMO Proteins

The first isoform of SUMO proteins was identified as sentrin in 1996 (18). Sentrin is demonstrated to form a covalent conjugate with proteins by attaching SUMO to specific lysine residue and regulate protein functions, suggesting a novel PTM mechanism (19, 20). Thus, SUMOylation is proposed to describe the modification process of various proteins via reversibly attaching to the lysine residues through a multi-step enzymatic reaction cascade (Figure 1).

To date, five SUMO isoforms (SUMO1, 2, 3, 4, and 5) have been identified (21). SUMO1 and 2/3 are widely distributed in the body, whereas SUMO4 and SUMO5 appear to be tissue-specific and less investigated though. The SUMO4 was only found in the kidney, spleen, and lymph nodes (9). What we know so far: SUMO4 is discovered as an intron-free pseudogene in the study of single nucleotide polymorphism in type 1 diabetes (22). As a recently discovered family member, SUMO5 was identified in primates and showed high tissue specificity and might be involved in the regulation of promyelocytic leukemia nuclear bodies (21, 23). Based on the primary structure, SUMO1 shows 48% sequence identity with both SUMO2 and SUMO3, while the latter two isoforms are highly similar and with 97% of identity (24). SUMO4 shares 85% identity with SUMO2/3 although no evidence supports the conjugation with substrates (4, 22). The SUMO5 is highly homologous to SUMO1 (25). On the other hand, SUMOylation occurs at selective lysine residue within the canonical/consensus motif of Φ-K-X-E/D, known as SUMO-interacting motif (SIM), where Φ is a hydrophobic residue, K is lysine for SUMO to form conjugate, X is any amino acid residue, and E/D is an acidic residue (26).

Nevertheless, SUMO isoforms have overlapping functions and may compensate each other under certain circumstances.

### Dynamic Balance Between SUMOylation and deSUMOylation

SUMOylation and deSUMOylation are dynamically balanced under physiological conditions. As shown in Figure 1, SUMOylation describes the covalent modification of protein substrates by SUMOs while deSUMOylation removes SUMOs from SUMOylated proteins. The SUMO proteases in the SUMO pathway are listed in Table 1. For forming covalent conjugates with protein substrates, SUMOs undergo maturation via proteolytic cleavage of the C-terminal Glycin -Glycin (~GG) dipeptide by SENP (28). SUMOs are attached to protein substrates through three reaction steps: ATP-dependent activation, SUMO- E2(UBC9) conjugation, and SUMO-protein substrate ligation. Firstly, the mature SUMO is activated by the E1 activating enzyme (SAE1/2) in an ATP-dependent manner. Secondly, the E2 conjugating enzyme UBC9 takes over SUMO moiety via forming covalent conjugate. Finally, the E3 ligase brings protein substrate and E2/UBC9 SUMO complex into the closer proximity and yields the covalent conjugate of the SUMOylated proteins including a protein inhibitor of activated STAT (PIAS) family, RanBR2, polycomb2, mitochondrial-anchored protein ligase (MAPL), and much other protein substrates (20, 29).

**Table 1:** Key enzymes in the SUMO pathway.

**Category**	**Name**	**Molecular weights**	**Cellular location**	**Isoform preference**	**Function**	**State of SUMO pathway**
SENPs	SENP1	73.5 kDa	Cytoplasm, nucleus	All SUMO	Maturation	SUMOylation
	SENP2	67.8 kDa	Cytoplasm, nucleus	All SUMO		
	SENP5	86.7 kDa	Nucleus (almost)	All SUMO		
SUMO E1	SAE1	38.5 kDa	Nucleus (almost)	All SUMO	Activation	SUMOylation
	SAE2	71.2 kDa	Cytoplasm, nucleus			
SUMO E2	UBC9	18.0 kDa	Nucleus (almost)	All SUMO	Conjugation	SUMOylation
SUMO E3	PIAS1	71.8 kDa	Nucleus (almost)	All SUMO	Ligation	SUMOylation
	PIAS3	68.0 kDa	Cytoplasm, nucleus			
	PIASy	56.5 kDa	Nucleus			
	RanBP2	358.2 kDa	Nucleus			
	MAPL	39.8 kDa	Mitochondrion			
	Pc2/CBX4	61.4 kDa	Nucleus			
	Smc5/6	128.8 kDa	Nucleus			
	ZNF451	121.5 kDa	Nucleus			
	Topors	119.2 kDa	Nucleus			
	ZIP3	33.6 kDa	Nucleus			
	Rhes	30.4 kDa	Cell membrane			
	KAP1	18.2 kDa	Cytoskeleton			
	NSE2	27.9 kDa	Nucleus			
	^*^SLX5/STUbLs	71.2 kDa	Nucleus			
	^*^SIZ1	100.8 kDa	Cytoplasm, nucleus			
	^*^SIZ2	81.2 kDa	Nucleus			
SENPS	SENP1	73.5 kDa	Cytoplasm, nucleus	All SUMO	Deconjugation	deSUMOylation
	SENP2	67.8 kDa	Cytoplasm, nucleus	All SUMO		
	SENP3	65.0 kDa	Nucleus (almost)	SUMO2/3		
	SENP5	86.7 kDa	Nucleus (almost)	SUMO2/3		
	SENP6	126.1 kDa	Nucleus (almost)	SUMO2/3		
	SENP7	119.7 kDa	Cytoplasm, nucleus**^#^**	SUMO2/3		

* *Specific for yeast while the others are present in human*.

# *In human cell, a majority of SENP7 was localized in nuclei whereas in mouse and rabbit cells, most SENP7 was distributed in the cytoplasm (27)*.

The deSUMOylation describes the removal of SUMO moiety from target proteins by the SENP and deSUMOylating isopeptidases (19, 30). The SENP family includes six members, namely SENP 1-3, 5-7 in humans. Different SENP members act on specific SUMOylated proteins (31). SENP1 and SENP2 catalyze the deSUMOylation of all types of SUMO proteins (32), whereas SENP3, 5, 6, and 7 prefer SUMO2/3-derived SUMOylated proteins (33, 34). Ultimately, deSUMOylation eliminates the effects of SUMOylation on protein functions (35).

SUMOylation plays important role in the various molecular events and processes (36). First, SUMOylation may promote or block the association of molecules that interact with SUMOylated substrates. Vivek et al. showed that SUMOylation mediated WRKY33 phosphorylation while disruption of WRKY33 SUMO sites inactivated WRKY33-mediated defense (37). Waizenegger et al. found that Mms4 was engaged by (poly)SUMOylation and targeted for proteasome degradation (38). Moreover, SUMOylation preserves substrate stability by competing with ubiquitination for the lysine residue or by recruiting the SUMO-targeted ubiquitin ligase (STUBL) family of proteins to the SUMOylated substrates. SUMOylation of hyperphosphorylated tau at K340 inhibits its ubiquitylation and the subsequent proteasome-dependent degradation (39). Gao et al. showed that PKCδ was SUMOylated at lysine 473, and the SUMOylation increased PKCδ stability by repressing its ubiquitination (40). Besides, SUMOylation induces conformational changes in proteins and thereby regulates protein functions (41–43). SUMOylation of PKM2 lysine 270 (K270) triggered conformation change from tetrameric to dimeric of PKM2, and reduced PKM activity (44). Furthermore, SUMOylation provides a new mechanism to recruit proteins with SUMO-interacting motif. Blondel et al. identified a non-covalent interaction between SUMO and β-arr2, via a SUMO interaction motif (SIM), that is, required for β-arr2 cytonuclear trafficking (45). Thus, SUMOylation and de-SUMOylation coordinate the regulation of various cellular signaling pathways (42).

### SUMOylation in Cardiovascular Disease

**Figure 2:**
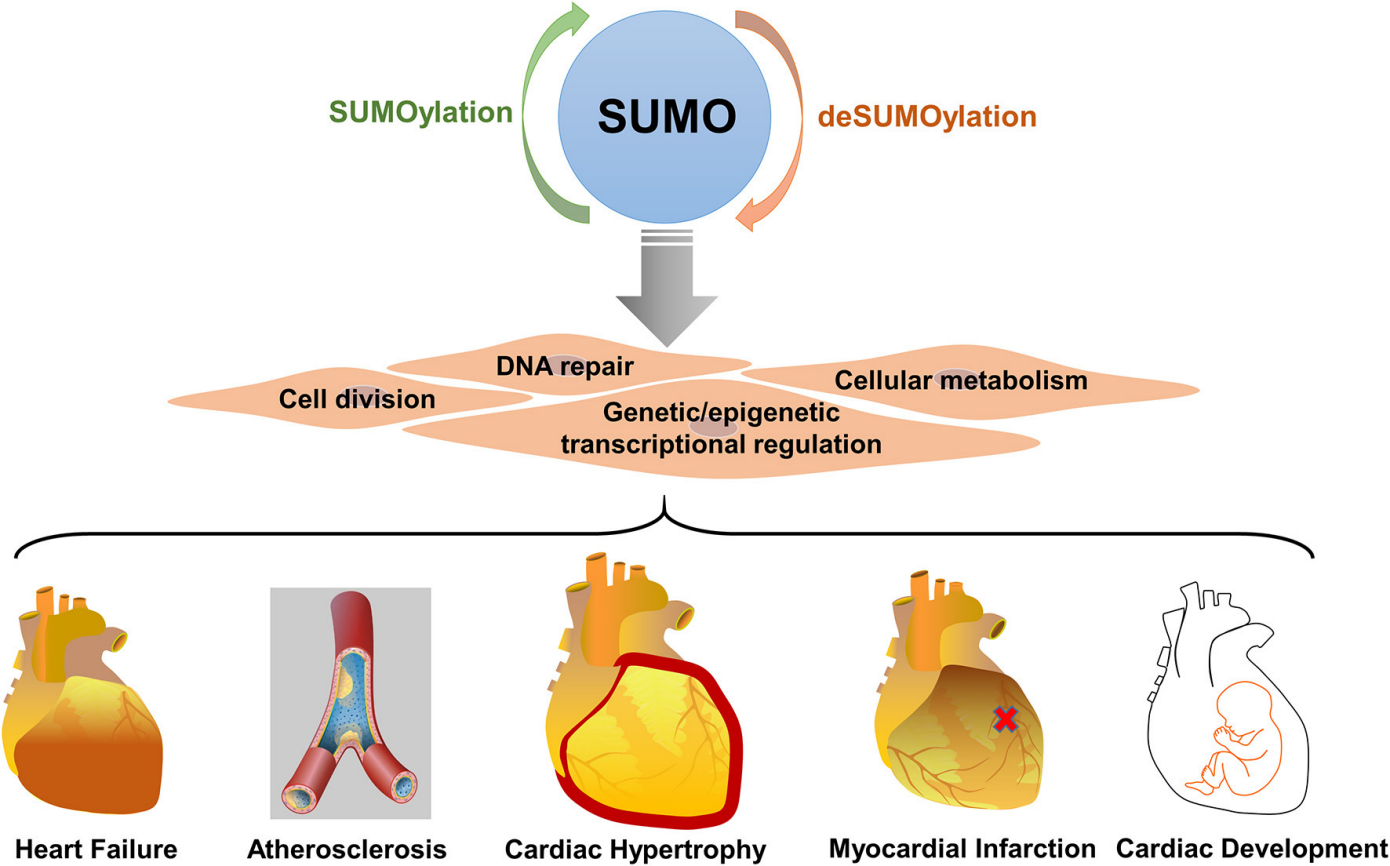
SUMOylation plays important role in cardiovascular disease. SUMOylation regulates multiple biological processes, including cell division, DNA repair, genetic and epigenetic transcriptional regulation, and cellular metabolism in the cardiovascular system. SUMOylation is dysregulated in heart failure, atherosclerosis, cardiac hypertrophy, myocardial infarction.

SUMOylation is an important PTM in the regulation of cell division, DNA repair, genetic and epigenetic transcriptional regulation, and cellular metabolism (4, 14, 20). Consequently, dysregulation of SUMOylation is closely related to the pathogenesis of various diseases, such as cancer, diabetes, epilepsy, and cardiovascular diseases (46, 47). Many recent studies suggest that SUMOylation is a therapeutic target in the cardiovascular system. Importantly, SUMOylation not only affects cardiac function and development (11, 48–50) but also controls the capacity of the heart to adapt to various pathological stresses (51) (Figure 2). Specific SUMO isoforms (e.g., SUMO1, 2, 3) are required and play a different role in the heart. SUMO-1 KO mice suffered from congenital heart diseases such as atrial and septal defects, progressive cardiac dysfunction and sudden death (52). Specifically, the over-expression of SUMO1 enhances cardiac function in mice with heart failure and increases contractility in isolated cardiomyocytes (10). SUMO-2 KO mice exhibited severe developmental delay, without any specific cardiac phenotype, and died at embryonic stage E10.5 whereas SUMO-3 KO mice were viable (53). Another study showed that the dynamics of the SUMOylation/deSUMOylation was altered during MI/R injury correlating with the decrease in SENPs activities, especially that of SENP3 (54). Presumably, the SUMOylation system may control the response of the heart to hypoxic/ischemic stress (55). Nevertheless, little is known about the regulation of the SUMOylation system and the potential target proteins during MI.

## SUMOylation in the Pathophysiological Process of Myocardial Infarction

MI occurs when blood supply to the heart is initially restricted. Upon the restoration of blood flow, the myocardial tissues receive concomitant reoxygenation. However, ischemia causes initial injury while reperfusion further exacerbates inflammatory responses in cardiac tissues (56). Thus, the ischemia-reperfusion injury consolidates the myocardial injury in two different pathophysiological states: ischemia and subsequent reperfusion (57–59).

### Ischemic State

Myocardial ischemia is caused by a decrease in coronary blood flow or failure to meet the demand of cardiac tissue for oxygen. Ischemia indicates that the environment oxygen is not enough to maintain the oxidative phosphorylation in the mitochondria (60). During ischemia, anaerobic glycolysis of glucose produces excessive lactic acid in the ischemic cells and ultimately causes acidification in the heart (61). When oxidative phosphorylation is not supported by sufficient oxygen, ATP production is largely reduced, leading to the dysfunction of Na^+^-K^+^-ATPase and the elevation of intracellular calcium, sodium, and hydrogen concentration (62, 63). Subsequently, cells swell while the activities of cytoplasmic enzymes are impaired. Prolonged ischemia causes progressive and irreversible injury in the heart. Morphologically, such irreversible injury is characterized by glycogen depletion, margination of nuclear chromatin, mitochondrial swelling, and sarcolemma breaks (64).

### Reperfusion State

Timely and complete reperfusion is critical to limit infarct size and subsequent ventricular remodeling (65). Reperfusion also causes irreversible injury to the myocardium and the coronary circulation, contributing to final infarct size (66, 67). During reperfusion, the mitochondrial permeability transition pore opening is triggered by multiple factors including mitochondrial calcium. Subsequently, oxidative stress and calcium overload increase the release of cytochrome C and thereby induce myocardial injury (68). Reperfusion not only salvages ischemic myocardium from infarction but also induces irreversible injury, leading to increased infarct size and microvascular dysfunction (69, 70).

Myocardial ischemia-reperfusion (MI/R) injury is a complex pathological process involving several signaling pathways (71, 72). As a key type of PTM, SUMOylation and deSUMOylation appear to affect different mechanisms toward cardiac damage. Several studies suggest that SUMOylation determines the fate of perfused hearts (73, 74). Ischemia increased SUMOylation levels, especially involving SUMO-2/3, while reperfusion further increased SUMOylation of various proteins in animal models and cell culture systems (75). For example, SUMO-2/3 conjugation was increased in failing human hearts (11). SUMOylation levels by SUMO-1 and SUMO-2/3 showed apparent differences in the mouse and rat models of ischemia-reperfusion injury (76, 77). In rats, a dramatic increase in SUMOylation by both SUMO-1 and SUMO-2/3 was observed at 6 h and 24 h in the striatal infarct area and hippocampus. In mice, no changes in SUMOylation occurred at 6 h but there was increased SUMO-1 conjugation in the cortical infarct after ischemia-reperfusion injury. Collectively, attention should be directed to the increase of SUMO2/3 conjugation in MI. Further work should be designed to clarify whether SUMOylation occurs mainly in the process of reperfusion or the stage of initial ischemia (41, 75, 78). Although the role of SUMOylation in MI/R injury remains elusive (54, 79, 80), SUMOylation may be a promising target for drug discovery against MI (30).

## SUMOylated Proteins in Myocardial Infarction

**Figure 3:**
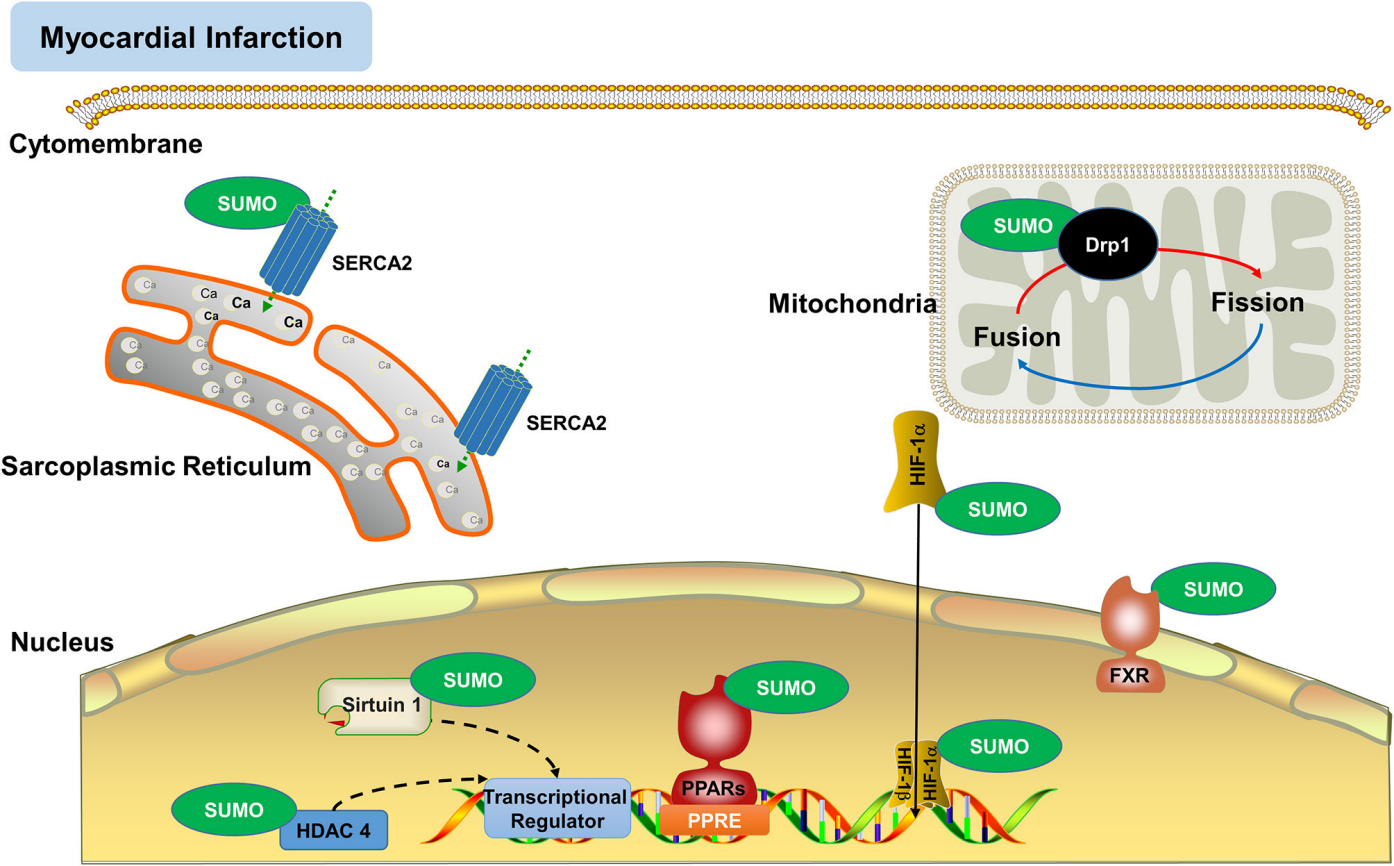
SUMOylation regulates the functions of various proteins in myocardial infarction. SUMOylation targets include various nuclear and extranuclear proteins (e.g., Drp1, HDAC4, HIF-1α, PPARs, SERCA2a, Sirtuin 1) in the regulation of cardiac functions and the prevention of reactive oxygen species (ROS) and myocardial injury.

Proteostasis is essential for maintaining cellular function, especially for the myocardial cell with low mitotic activity. PTM is well-known to modulate protein function and fate, suggesting that PTM plays an essential role in proteostasis (81). It is not surprising that SUMOylation positively contributes to heart function and proteostasis. SUMO isoforms are functionally distinct and modify different substrates. SUMOylation by different SUMO isoforms may exhibit unique subcellular localization patterns and dynamics (4, 20). Although most of the SUMO proteins are present in the nucleus, SUMOylation also occurs on extra-nuclear proteins (42). Consequently, SUMOylation regulates protein functions in intro-cellular trafficking, apoptosis, protein stability, and enzyme activity (9, 33). SUMOylation and deSUMOylation coordinately affect the protein levels and thereby control the extent of MI/R injury (30). The SUMOylation of proteins is regulated by multiple signaling pathways toward the modulation of cardiac functions and development (20, 82–84). Other work also indicated that SUMOylation targeted the proteins that were implicated in ischemic heart disease (50). For example, SUMOylation targeted various proteins including peroxisome proliferator-activator receptors (PPARs) (85), silent information regulator (Sirtuin) 1(86), histone deacetylase (HDAC) 4, hypoxia-inducible factor (HIF)-1α (87), sarcoplasmic/endoplasmic reticulum Ca^2+^ ATPase2a (SERCA2a) (8, 48), and dynamin-related protein (Drp) 1(88). As SUMOylation plays different roles in the nucleus and the cytoplasm. The location and heart-specific functions of SUMOylated proteins are discussed in Figure 3. SUMOylation of proteins may alleviate or exacerbate myocardial damage for potential inhibitory or promoting effects (30, 80, 89). It is well-known that SUMOylation occurs at the specific sites of target proteins. Thus, it is particularly important to identify SUMOylation sites for the development of new drugs. We employed online software GPS-SUMO (http://sumosp.biocuckoo.org/) to analyze the SUMOylation target proteins that were previously reported for direct relatedness to myocardial infarction. The predicted SUMOylation sites of the selected protein targets are listed in Table 2.

**Table 2:** List of the predicted SUMOylation sites^*^.

**Protein symbol**	**Uniprot ID**	**Sub-organelle localization**	**SUMOylation sites**	***P*-value**	**Pathophysiological function**
PPAR-γ	P37231	nuclear compartment	QEYQSAI**K**^107^VEPASPP FGDFMEP**K**^395^FEFAVKF	0.000 0.037	Suppress apoptotic and inflammatory injury
HDAC4	P56524	nuclear compartment	AQAGVQV**K**^559^QEPIESD	0.001	Enhance the survival of cardiomyocytes
SIRT1	Q96EB6	nuclear compartment	INEAISV**K**^734^QEVTDMN DEPLAIM**K**^408^PEIVFFG EQFHRAM**K**^427^YDKDEVD DMNYPSN**K**^746^S	0.004 0.014 0.042 0.049	Enhance the myocardial cell Adaption to genotoxic stress; Regulate cardiac metabolism
FXR	P51114	nuclear compartment	LPPPPDI**K**^56^KEISEGD EEKINTL**K**^607^EENTQEA LAKVRMM**K**^88^GEFYVIE	0.005 0.006 0.026	Modulate the adverse cardiac effects of FXR
HIF-1α	Q16665	nuclear compartment	SSLFDKL**K**^391^KEPDALT LNQEVAL**K**^477^LEPNPES SDMVNEF**K**^532^LELVEKL	0.002 0.003 0.026	Lead to the degradation of HIF-1α
SERCA2a	P16615	sarcoplasmic reticulum membrane	SVIKQLM**K**^480^KEFTLEF EDSANFI**K**^585^YETNLTF	0.004 0.005	Enhance intracellular mitochondrial membrane potential and reduce cell apoptosis
Drp1	O00429	mitochondria	RGMLKTS**K**^597^AEELLAE	0.072	Increase mitophagy; prevent ROS generation and cell death

* *The prediction was carried out by Software GPS-SUMO (http://sumosp.biocuckoo.org/)*.

### SUMOylated Proteins in the Nuclear Compartment

#### Peroxisome Proliferator-Activated Receptor Isoforms-γ

Peroxisome proliferator-activated receptors (PPARs) are ligand-activated transcription factors of the nuclear hormone receptor superfamily including three subtypes: PPAR-α, PPAR-β/δ, and PPAR-γ (90). Cellular energy expenditure is tightly controlled by the PPAR family of transcription factors exclusively in the nucleus (91). Especially, PPAR-γ plays an important role in various physiological and pathological processes, including glucose and lipid metabolism, immunity (92). PPAR-γ is shown to be beneficial in the treatment of cardiovascular diseases such as those related to ventricular hypertrophy, cardiac remodeling, and acute myocardial infarction (93, 94). Activation of PPAR-γ suppresses the inflammatory response in cardiac tissue after ischemia-reperfusion and alleviates pathological ischemic damage (95).

The activities of PPARs and associated co-regulators are regulated by PTMs, such as ubiquitylation and SUMOylation in different cell types. Presumably, SUMOylation regulates the roles of PPARs in cardiomyocytes (96, 97). SUMOylation of PPAR-γ at lysine 77 in the transactivation domain blocks its transcriptional activity by promoting co-repressor recruitment (98). PPAR-γ is also SUMOylated at lysine 365, which causes PPAR-γ to occupy the promoters of inflammatory genes and inhibit their expression in macrophages (99). The protein inhibitor of activated STAT1 (PIAS1) is a specific E3 ligase for PPAR-γ SUMOylation in the myocardium. During the ischemia-reperfusion process, PIAS1 was down-regulated so that the PPARγ SUMOylation was accordingly reduced. Such decline in PPARγ SUMOylation caused dysregulation of NF-κB activity and suppression of anti-apoptotic and anti-inflammatory activities, leading to exacerbation of ischemia-reperfusion injury (17).

#### Histone Deacetylase 4

Histone deacetylases (HDACs) constitute a family of transcriptional regulators that catalyze an important type of PTM in cardiovascular diseases. HADCs inhibitors have been reported to have a cardioprotective function partially due to the promotion of protein SUMOylation in cardiomyocytes and fibroblasts (100). An earlier study demonstrated that HDACs may promote the production of reactive oxygen species (ROS) and mitochondrial damage in the period of reperfusion (101). HDAC4 belongs to class II a group of HDACs which is an important regulator of gene expression that controls pleiotropic cellular functions (102). Previous research indicated that HDAC4 SUMOylation elicited the degradation of HDAC4, caused the suppression of HDAC activity and interrupted the cellular protective pathway (103). Another study showed that SUMOylation of HDAC4 could enhance the survival and reduce apoptosis of cardiomyocytes by decreasing lactate dehydrogenase (LDH) leakage, ratio of caspase-3-positive cells in hypoxia-reoxygenation injury (103). Interestingly, SUMOylation of HDAC4 may also reduce the production of ROS and mitochondrial dysfunction and provide indirect protection to cardiomyocytes (30). Irisin as a recently discovered cardio-myokine could decrease the protein levels of HDAC4 and alleviate the MI/R injury through SUMOylation-dependent mechanisms (104). Thus, drugs are needed to promote HDAC4 SUMOylation against MI/R injury.

#### Silent Information Regulator 1 (Sirtuin 1)

Silent information regulator 1 (Sirtuin 1), a nicotinamide adenine dinucleotide (NAD)-dependent deacetylase, regulates gene expression by histone deacetylation. Recent studies demonstrated that Sirtuin 1 played an intricate role in the pathology, progression, and treatment of several diseases (105, 106). SUMOylation promoted the deacetylase activity of Sirtuin1 and enhanced the cell adaption to genotoxic stress (86). Cardiac Sirtuin1 is mainly expressed and SUMOylated in cardiomyocyte nuclei, supporting myocardial tolerance to ischemia (107). Moreover, SUMOylation promotes Sirtuin 1 activation in ischemic preconditioning (108). The SUMOylation not only modifies Sirtuin 1 to be a cardioprotective and adaptive factor under different cardiac stress conditions but also reduces the apoptosis of myocardial cells (109–112). Yang et al. showed that reduction of Sirtuin 1 SUMOylation in response to DNA damage attenuated its HDAC activity, enhanced the activity of its pro-apoptotic substrates and ultimately resulted in cell death (86). ROS caused the decline of Sirtuin 1 activity and disrupted the resistance to MI/R injury in the aged mice, possibly via affecting SUMO1 and deSUMOylase activity in the heart (113). Future work should be directed to investigate whether SUMOylation regulates the nuclear vs. cytoplasmic localization of Sirtuin 1(112) and identify which of SUMO1- and SUMO2/3 dominates the SUMOylation of Sirtuin1 in the heart.

#### Farnesoid-X-Receptor

Farnesoid-X-receptor (FXR), a nuclear hormone receptor, is abundantly expressed in the liver and gastrointestinal tract and plays crucial roles in the metabolism of lipids, cholesterol, bile acid, and glucose (114, 115). It was previously reported that FXR was also expressed in the heart and mediated pro-apoptotic cell signals in MI/R injury (116). FXR may be SUMOylated in cardiac tissue while the level of FXR SUMOylation is down-regulated during ischemia and reperfusion (117). Such decline of FXR SUMOylation increased FXR transcription activity and subsequently upregulated the expression of FXR target gene SHP, leading to intrinsic apoptosis and autophagy impairment during IR injury (118). The mutation of the SUMOylation site in the FXR sequence weakened the SUMOylation while enhanced the adverse effects of FXR in MI/R injury (118). Meanwhile, the activation of the mitochondrial apoptotic pathway and the dysfunction of the autophagy pathway were observed (118). Thus, SUMOylation is an important pathway to modulate the adverse cardiac effects of FXR in myocardial infarction.

#### Hypoxia-Inducible Factor-1 α

Hypoxia-inducible factor (HIF) plays an essential role in cellular and systemic oxygen homeostasis via inducing the expression of many hypoxia-responsive genes (119). HIF-1α mediates hypoxia-signaling cascade to exhibit myocardial protection in MI/R (120). HIF-1α could be SUMOylated by SUMO1, SUMO2/3 during hypoxia (121). Such changes might affect several critical regulatory pathways in mammalian cells. Interestingly, SUMO1 mediated the SUMOylation of HIF-1α at Lys391 and Lys477 residues, which stabilized the protein and promoted the transcriptional activity of HIF-1α during hypoxia (122). Indeed, SUMO1 and HIF-1α were co-expressed and formed complex in cardiomyocytes during hypoxia stimulation (123). HIF-1α is well-known to protect the heart against IR injury (124). SENP1 deconjugates SUMOylated HIF-1α and prevents HIF-1α degradation during hypoxia. SENP1 and SENP3 catalyze deSUMOylation of HIF-1α and thereby exhibit cardio-protection (125). SENP1 was up-regulated during ischemia and reperfusion, and thereby activated the HIF-1α pathway, and supported its cardioprotective role (126). Cheng et al. showed that the protein level of HIF-1α was significantly decreased in SENP1^−/−^ mouse embryonic cells while HIF-1α target genes including VEGF and GLUT-1 were down-regulated (87). Other studies confirmed that SENP1 deficiency exacerbated myocardial injury in the experimental MI/R model via the HIF-1α-dependent mechanism (87, 126). These results revealed an essential physiological role of SENP1 in the hypoxia response through the regulation of HIF-1α stability (87).

### SUMOylated Proteins on Sarcoplasmic Reticulum Membrane

Sarcoplasmic reticulum (SR) Ca^2+^ ATPase pump (SERCA2a) is an important ATP hydrolase and highly expressed in the heart for the control of Ca^2+^ reuptake and replenishment to SR in excitation-contraction coupling (30). Phospholamban phosphorylation increases the ATPase activity of SERCA2a and enhanced Ca^2+^ transport into the endoplasmic reticulum for the next round of the contraction cycle (127). Dysregulation of SERCA2a activity and Ca^2+^ cycling hallmark the pathology of heart failure and may drive the development of other cardiac dysfunctions (128). A recent study found that SUMOylation of SERCA2a increased the expression and activity of SERCA2a partly through SUMO1, thus improving ΔΨm and reducing apoptotic cells *in vitro* and promoting the recovery of heart function and reducing infarct size *in vivo* (129). Mouse SERCA2a could be SUMOylated at the sites of lysine 585, 480 and 571, whereas SUMOylation at lysine 585 enhanced SERCA2a stability (129, 130). The protein levels of SERCA2a, SUMO1, and the SUMOylated SERCA2a (S-SERCA2a) were decreased in myocardial injury (14). Interestingly, SUMO1 knockdown exacerbated while SUMO1 overexpression reversed the decline of SERCA2a function and SUMOylated SERCA2a (10, 129). Moreover, SUMO1 overexpression decreases cardiomyocyte apoptosis, reduces infarct size, and increases cardiac function (48). Collectively, SUMO1 mediated-SUMOylation of SERCA2a appears to be an important cardioprotective mechanism against MI/R injury (129).

### SUMOylation of Mitochondrial Protein Dynamin-Related Protein 1

Mitochondrial dysfunction is implicated in MI/R injury (131, 132). Dynamin-related protein (Drp) 1 is an important mitochondrial protein in the regulation of mitochondrial morphology and fission (15). It was reported that SUMOylated Drp1 was the key to mediate zinc-induced cardioprotection against I/R injury, possibly via activating the mitophagy of the mitochondria and suppressing ROS production (133). SUMOylation might inhibit Drp1 translocation from the cytosol to mitochondria, maintained the mitochondrial morphology, restrained mitochondrial fission, and protected the heart against MI/R injury (134). Interestingly, SUMOylation of Drp1 prevents MI/R injury, suggesting a protective mechanism against stress (80). SUMOylation of Drp1 increased mitophagy during reperfusion, leading to the prevention of ROS, myocardial apoptosis, and myocardial injury (135). With the increase in SUMO1-mediated SUMOylation of Drp1, the mitochondrial translocation of Drp1 is prevented (15). MI/R appears to increase the expression of Drp1 in mitochondria and the SUMOylations of Drp1 by both SUMO1 and SUMO2/3 (74).

On the other hand, the biological functions of SUMOylated proteins are reversed by deSUMOylation. For deSUMOylation, SENP2 removes SUMO1 from Drp1 whereas SENP3 and SENP5 remove SUMO2/3 from Drp1 (74, 136). SENP3 and SENP5 preferentially remove SUMO2/3 from Drp1 and inhibits SUMO2/3-induced mitochondrial translocation of Drp1(137). Pharmacological inhibition of Drp1 by mitochondrial division inhibitor-1(Mdivi-1) significantly attenuated the effects of SENP3 on mitochondrial membrane potential, mitochondrial swelling and cardiac injury (54). Others found the decrease in SUMO2/3-mediated SUMOylation of Drp1 and the reduction of mitochondrial fission level, ROS generation, and cell death in SENP5 transgenic mice (49). Collectively, Mdivi-1 may protect the myocardium by inhibiting the SENP3 mediated-deSUMOylation pathway. These studies suggest that the balance of SUMOylation and deSUMOylation may be a potential therapeutic target for the treatment of MI.

## SUMOylation as a Potential Therapeutic Target

The existing clinical therapies do not fully resolve MI/R injury in patients with coronary heart disease. The mechanism of SUMOylation has not yet been reported in clinical therapies such as vascular interventional and drug thrombolysis. But it would be a good future direction. Some basic studies have investigated the potential of SUMOylation in the therapy of MI/R injury. As listed in Table 3, indeed, several physical methods and chemical compounds are under development to target the SUMO pathway. Moderate hypothermia significantly enhanced SUMO1-mediated SUMOylation of various target proteins in cardiomyocytes (138). These results suggested that moderate hypothermia significantly increased SUMO1 and Bcl-2 expression levels, as well as the mitochondrial membrane potential, but significantly decreased the expression levels of caspase-3 and mitochondrial ROS. Thus, moderate hypothermia may enhance SUMOylation and attenuate myocardial H/R injury (138). SUMOylation of Drp1 regulated mitochondrial autophagy and promoted the protective effect of zinc on hypoxia-reoxygenation injury (80). During the MI/R injury, SUMO1 conjugation was inactivated while Zinc induced mitophagy via increasing Drp1 SUMO1-mediated SUMOylation to clear damaged mitochondria, control mitochondrial quality, and prevent ROS generation, ultimately reducing MI/R injury (80).

**Table 3:** Anti-MI Therapeutics via targeting SUMOylation.

**Therapeutics/regents**	**SUMOylated protein/substrate**	**SUMO proteases**	**Function**	**Phase of research**	**References**
Moderate hypothermia	/	SUMO1	enhancing SUMOylation and attenuating MI/R injury	Clinical trial	(123)
Zinc	Drp1	/	Regulating mitochondrial autophagy and reducing MI/R injury	Preclinical study	(67)
Luteolin	SERCA2a	SUMO1	Stabilizing SERCA2a	Preclinical study	(114, 115)
Ginkgolic acid	PML/Pin1/TGF-β1	SUMO1	Inhibiting cardiac fibrosis	Preclinical study	(124)
Irisin	HDAC4	SUMO1	Improving degradation of HDAC4	Preclinical study	(91)
Mdivi-1	Drp1	SENP3	Attenuating mitochondrial abnormality and cardiac injury	Preclinical study	(65)
TAK-981	/	SUMO molecule	Inhibiting SUMOylation	Phase 1 clinical trial	(29)
PIAS1	PPAR-γ	SUMO E3 ligases	Inhibiting apoptosis and inflammation	Preclinical study	(86)

Naturally occurring bioactive components are identified from traditional Chinese medicine for further evaluation. As an example, luteolin is a plant flavonoid with profound antioxidant and immunomodulatory properties (139, 140). Pharmacological studies demonstrated that luteolin not only increased the phosphorylation of protein kinase B (Akt) and phospholaban (PLB) but also the sumoylation of SERCA2a, and specificity protein 1 (Sp1) (130). In addition, luteolin upregulated the expression ratio of Bcl-2/Bax, caspase-3/cleaved-Caspase3(130). A recent study demonstrated that luteolin stabilized the binding of SUMO1 to SERCA2a and preferably promoted SUMO1-mediated SUMOylation of SERCA2a at the K585 site (130). Another study revealed that SUMOylation of SERCA2a was a key mechanism to support the cardioprotective activity of luteolin against MI/R injury (129). As a phenolic acid from the plant Ginkgo biloba L, ginkgolic acid inhibited protein SUMOylation by blocking the formation of the E1-SUMO1 intermediate and thereby prevented cardiac fibrosis in myocardial infarction via inhibiting SUMO-1(141). On the other hand, as a recently discovered protein hormone, irisin is initially synthesized in muscles in response to exercise for regulating metabolism and energy expenditure (142, 143). It was found that irisin exhibited cardioprotective effects in myocardial hypoxia-reoxygenation *via* SUMOylation mediated-HDAC4 degradation (104).

Synthetic small molecules represent another category for the evaluation as MI therapy. PIAS1 is known as a specific E3 ligase for PPAR-γ SUMOylation and reduces apoptotic and inflammatory injury by inhibiting NF-κB pathway after ischemia/reperfusion (17). Mdivi-1, a pharmacological inhibitor of Drp1, significantly attenuated the mitochondrial abnormality and cardiac injury in the model of overexpressing SENP3 via inhibiting Drp1 after MI/R injury (54). Moreover, the effort is made to evaluate the Drp1 inhibitor for preventing atherosclerosis in a clinical trial [ClinicalTrials.gov Identifier: NCT03980548] (33). TAK-981 inhibits the transfer of SUMO protein from E1 to E2 by binding to the C-terminal of SUMO proteins (144). Consequently, TAK-981 is tested in patients with neoplasms or lymphomas in phase 1 clinical trial [ClinicalTrials.gov Identifier: NCT03648372] (145). Collectively, as a key PTM, the potential SUMOylation should be fully explored for the development of new therapies against myocardial infarction.

## Conclusion

This review discussed the effects of SUMOylation on two key pathophysiological stages of MI. The SUMO proteins modify many proteins involved in different signaling pathways so that some SUMOylated proteins exhibit different expression levels and functions against MI. When more studies are available, it would be important to study the cross-talk between SUMOylation and other types of PTM. The current understanding of SUMOylation in MI is mainly based on the previous studies in the early stage of reperfusion. Future work should elucidate the SUMOylation in cardiomyocytes at the remodeling phase after reperfusion. It is positive that SUMOylation is a promising therapeutic target for the treatment of myocardial infarction.

## Author Contributions

The manuscript was designed by WZ and JR. WZ wrote the first draft of the manuscript. JR and XZ revised the manuscript. All authors contributed to the article and approved the submitted version.

## Conflict of Interest

The authors declare that the research was conducted in the absence of any commercial or financial relationships that could be construed as a potential conflict of interest.

## Publisher's Note

All claims expressed in this article are solely those of the authors and do not necessarily represent those of their affiliated organizations, or those of the publisher, the editors and the reviewers. Any product that may be evaluated in this article, or claim that may be made by its manufacturer, is not guaranteed or endorsed by the publisher.

## Abbreviations

MI: myocardial infarction
SUMO: small ubiquitin-like modifier
PTM: post-translational modifications
SENP: sentrin-specific protease
MI/R: myocardial ischemia-reperfusion
PPAR: peroxisome proliferator activated receptor
HDAC: histone deacetylase
Sirtuin 1: silent information regulator 1
HIF-1α: hypoxia-inducible factor−1α
Drp 1: dynamin-related protein1
SERCA2a: sarcoplasmic reticulum Ca^2+^ ATPase pump
FXR: farnesoid-X-receptor
PIAS1: protein inhibitor of activated STAT1.
